# Elective minimally invasive coronary artery bypass: Shunt or tournique occlusion? Assessment of a protective role of perioperative left anterior descending shunting on myocardial damage. A prospective randomized study

**DOI:** 10.1186/1749-8090-7-69

**Published:** 2012-07-18

**Authors:** Zdenek Sorm, Jan Vojacek, Eva Cermakova, Radek Pudil, Ulrich A Stock, Jan Harrer

**Affiliations:** 1 Department of Cardiac Surgery, Charles University in Prague, Faculty of Medicine in Hradec Kralove, University Hospital Hradec Kralove, Sokolska 581, 50005, Hradec Kralove, Czech Republic; 2 Eva Cermakova, Technology Center, Charles University in Prague, Faculty of Medicine in Hradec Kralove, Simkova 870, 500 38, Hradec Kralove, Czech Republic; 3 Radek Pudil, 1st Department of Medicine, Charles University in Prague, Faculty of Medicine in Hradec Kralove, University Hospital Hradec Kralove, Sokolska 581, 50005, Hradec Kralove, Czech Republic; 4 Ulrich Alfred Stock, Department of Thoracic, Cardiac and Vascular Surgery, University Hospital Tübingen, Tübingen, Germany

**Keywords:** MIDCAB, Intraluminal shunt, External tournique occlusion, Myocardial damage

## Abstract

**Background:**

To determine impact of intraluminal-left anterior descending shunt to prevent myocardial damage in minimally invasive coronary artery bypass.

**Methods:**

38 patients were randomly assigned to external tournique occlusion (n = 19) or intraluminal-left anterior descending shunt group (n = 19). Blood samples for cardiac troponin T were collected at 30 minutes prior to, 6 and 24 hours after surgery.

**Results:**

One patient in external tournique occlusion and two patients in intraluminal-left anterior descending shunt group were excluded from futher analysis due to preoperative cardiac troponin T level above the 99th-percentile. Postoperatively, each six patients in external tournique occlusion (33.3%) and intraluminal-left anterior descending shunt (35.3%) group were above the 99th-percentile. Two patients from each group (external tournique occlusion group 11.1% vs. intraluminal-left anterior descending shunt group 11.8%) had peak values above 10-% coeficient of variation cutoff (*p* = 1). There were no significant differences in between both groups at all studied timepoints.

**Conclusion:**

There was no protective effect of intraluminal shunting on myocardial damage compared to short-term tournique occlusion. It is upon the surgeon's discretion which method may preferrably be used to achieve a bloodless field in grafting of the non-occluded left anterior descending in minimally invasive coronary artery bypass.

## Background

Minimally invasive direct coronary artery bypass grafting (MIDCAB) is an excellent option for treatment of the patients with isolated stenotic or occluded left anterior descending coronary arteries (LAD) and selected patients with multivessel disease [1]. MIDCAB limits the patient burden and provides excellent long-term patency [2]. Bloodless operating field remains one of the most important prerequisite to perform perfect coronary anastomosis on the beating heart, especially in the case of the limited skin incision via left anterior mini-thoracotomy. Tournique occlusion or shunting of the target artery are two established methods to achieve this condition. External tournique occlusion (TO) of the LAD may cause transient perioperative anterior wall dysfunction [3,4], postoperative myocardial stunning [5,6] or increasing serum concentrations of cardiac troponin (cTn) [7]. Increased postoperative cardiac markers correlate with an impaired outcome after coronary artery bypass grafting (CABG) [8]. Intraluminal-LAD shunt (ILS) has proven to preserve segmental wall motion contractility during anastomoses by maintaining myocardial perfusion [9], reduces myocardial damage [7], shows a trend towards improved intra- and postoperative angiografic results [4], improve visualization and allow surgeons to perform the anastomosis in an unhurried and technically precise manner [10].

Clinical and histopathological safety of tournique occlusion or intraluminal shunt technique remains controversial [11-15]. The aim of this study was to evaluate the benefit of intraluminal-LAD shunt on myocardial damage in MIDCAB patients.

## Methods

### Patients

From April 2005 to August 2009 145 MIDCABs were performed at Department of Cardiac Surgery, Charles University in Prague, Faculty of Medicine and University Hospital Hradec Kralove, Czech Republic.

Of those, 38 patients met inclusion criteria and were randomly assigned to a external tournique occlusion group (TO group; n = 19) or a intraluminal-LAD shunt group (ILS group; n = 19). All patients included in the study were operated by the same supervised trainee and by the same consultant. The study was approved by the local ethics committe, informed consent was obtained from all patients. The exclusion criteria for the study were history of occlusion or myocardial infarction in the LAD territory, concomittant occlusion of both circumflex artery and right coronary artery, impaired left ventricular ejection fraction (LV EF) of less than 25% assessed by echocardiogram, redo-surgery, emergency operations, recent myocardial infarction (MI): < 3 weeks, renal insuficiency with serum creatinine level of more than 200 ug/L, age of < 18 years and of > 80 years.

The reasons for incomplete revascularization in 10 patients with double vessel disease were very small diameter of target vessels < 1.0-mm (7 patients) and intended percutaneous coronary intervention (PCI) on the other coronary artery after recovery from the operation (3 patients). Decision to perform hybrid MIDCAB procedure was made preoperatively by the heart team (cardiac surgeon and cardiologist).

### Anesthetic and surgical technique

Anesthetic technique consisted of propofol infusion at 1 mg/kg/h combined with sufentanyl infusion at 0,5 μg/kg/h. Neuromuscular blockade was achieved by 0.04 mg/kg/h cisatracurium. Patients were normocapnic ventilated using isoflurane (Forane, Abbot Laboratories, GB; 0,7–0,8%). Betablockers were applicated throughout the cardiac procedure. A left anterior minithoracotomy was made in the 4^th^ or 5^th^ intercostal space (length of the incision was at an average 8 centimeters). Left internal thoracic artery (LITA) was harvested under direct vision as a pedicled conduit. The patients were heparinized (100 IU/kg); the activated clotting time (HEMOCHRON® International Technidyne Corporation, Edison, USA) was kept at 300 seconds throughout the operation and was neutralized incompletely with a half dose of Protamine after completion of the anastomosis. Once the target region for the anastomosis on the LAD was identified, silicon loops in a figure-of-eight (Quest Medical, Inc, Allen, TX) were placed proximally in all patients. Very gentile stabilization of the heart with a mechanical stabilizer (CardioThoracic System, Inc, Cupertino, CA or StableSoftTM Ultra Stabilizer, Estech) was done. The proximal silicon loop was slightly elevated to avoid excessive bleeding during introduction of the intraluminal shunt after arteriotomy in the ILS group. The size (1.25, 1.5, 1.75 mm) of the shunt (Guidant Axius, Boston Scientific, Santa Clara, CA) were determined according to the target vessel diameter by the surgeon, oversizing was avoided. The shunt was removed prior to completion of the anastomosis. In the TO group ischemic preconditioning consisting of 3 minutes (min.) of ischemia and 5 min. of reperfusion was performed. The proximal tournique occlusion was maintained throughout completion of the anastomosis with running 7–0 polyprolylene suture (Prolen, Visi-black, Ethicon) in both groups. Excellent visibility was accomplished with a surgical blower (Axius^TM^ Blower/Mister, Maquet Cardiovascular LLC, Wayne, NJ). The open left pleural cavity was drained in all patients and the chest wall sutured in layers. To prevent chest pain after the operation, an intercostal nerve blockade was applied using 20 cc 0.5% bupivacaine hydrochloride (Solupharm GmbH, Melsungen, Germany) infiltration.

### ECG

All patients had a twelve lead electrocardiogram (ECG) assessment prior surgery, daily on intensive care unit (ICU) and at discharge. ECGs were independently analyzed by two observers who were blinded to the subsequent groups and outcome.

For intraoperative ECG on-line monitoring (Draeger Medical Inc., Telford, USA) with electrodes placed in the standard fashion was performed. ST-segment changes in lead V5 were included in the analysis.

### Cardiac troponin T

Three samples of central venous blood in each patient were analysed: 30 min. prior to surgery (baseline), 6 and 24 hours after the operation. The highest cTnT value during first 24 hours after operation was considered as peak cTnT serum concentration in each patient and was included in the analysis. cTnT was measured with an Elecsys 2010 Systems analyser (Roche Diagnostics GmbH, Mannheim, Germany) with current commercially available cTnT immunoassay (fourth generation). This has lower limit of detection (LLD) of 0.01 μg/L, a coeficient of variation (CV) ≤10% at 0.03 μg/L, and a 99th percentile cutoff at 0.01 μg/L.

### Criteria for myocardial necrosis and for CABG-related myocardial infarction (MI)

The cutoff for myocardial necrosis was determined to be 0.03 μg/L using the 10% total imprecision CV criteria (10-% CV cutoff).

Increases of cTnT serum concentration greater than quintuple of 10-% CV cutoff was determined as CABG-related MI [8].

### Statistical considerations

Statistical analysis was performed using the NCSS® 7 software at the 5% statistical significance level. The categorical data was analyzed by the Fisher's exact test (including cTnT analysis). The Mann–Whitney *U*-test, Kolmogorov-Smirnov and the Student unpaired *t*-test were used for the quantitative data. The variables in tables are expressed as the average ± standard deviation (SD) or as the median; 95% lower confidence limit (LCL) – 95% upper confidence limit (UCL).

## Results

One patient from TO group and two patients from ILS group were excluded from further analysis due to increased preoperative cTnT levels (above the 99th-percentile [0.01 μg/L]).

### Patients characteristics

Clinical and angiographic characteristics are depicted in Table 1. There were no significant differences between groups undergoing MIDCAB.

**Table 1:** Preoperative Comorbidity and Risk Factors

**Randomization**	**TO group, n = 18**	**ILS group, n = 17**	***p*-value**
Age*, years	62.9 ± 11.2	68.2 ± 9.2	0.14
Body mass index*	28.3 ± 3.9	29.8 ± 3.1	0.20
Female, n	4 (22.2)	2 (11.8)	0.66
Cerebrovascular disease, n	1 (5.6)	4 (23.5)	0.18
Diabetes mellitus, n	2 (11.1)	3 (17.6)	0.79
Previous myocardial infarction, n	6 (33.3)	4 (23.5)	0.71
Percutaneous coronary intervention, n	8 (44.4)	6 (35.3)	0.73
Peripheral vascular disease, n	1 (5.6)	0 (0)	1.00
History of unstable angina pectoris, n	5 (27.8)	2 (11.8)	0.40
Smoking, n	9 (50)	10 (58.8)	0.74
Atrial fibrillation, n	3 (16.7)	2 (11.8)	1.00
Left main stenosis, n	0 (0)	0 (0)	1.00
1-vessel disease, n	13 (72.2)	12 (70.6)	1.00
2-vessel disease, n	5 (27.8)	5 (29.4)	1.00
LAD stenosis			
50–70%, n	3 (16.7)	3 (17.6)	1.00
> 70%, n	15 (83.3)	14 (82.4)	1.00
Beta-blocker administration, n	16 (88.9)	12 (70.6)	0.23
Acetyl salicylic acid administration, n	1 (5.6)	2 (11.8)	0.60
Ejection fraction†, %	62; 60–65	65; 60–70	0.34
EuroSCORE†	2.08; 1.31–2.69	2.21; 1.33–2.69	0.99
Hypertension, n	14 (77.8)	14 (82.4)	1.00
Hyperlipidemia, n	13 (72.2)	10 (58.8)	0.49
Chronic obstructive pulmonary disease, n	0 (0)	0 (0)	1.00
Serum creatinin level*, mmol/L	80.6 ± 17.4	92.2 ± 16.5	0.051

* Values are expressed as mean ± SD.

† Values are expressed as median; 95%LCL-95%UCL.

Values in parentheses are percentages.

TO = tournique occlusion; LAD = left anterior descending; ILS = intraluminal-LAD shunt.

EuroSCORE = the European system for cardiac operative risk evaluation; SD = standard deviation; LCL = lower confidence limit; UCL = upper confidence limit.

### Intra-operative results

There were no conversion to sternotomy in our patients. The anastomotic time in TO group was significantly shorter than in ILS group (13.44 ± 5.06 vs 18.9 ± 6.56 min., *p* = 0.0094).

Intraoperative reversible ST segment deviations in precordial lead V5 were comparable in both groups (TO group: median 0.1 mV, range 0–1.4 mV vs ILS group: median 0.1 mV, range: 0 – 1.1 mV, *p* = 0.69). Intraoperative date are summarized in Table 2.

**Table 2:** Perioperative data (hemodynamic data and peak ST segment shift)

	**TO group, n = 18**	**ILS group, n = 17**	***p*-value**
Anastomosis time*, min.	13.4 ± 5.1	18.9 ± 6.6	0.0094
Heart rate†, beats/min.	58.5; 57–62	60; 57–60	0.84
Systolic blood pressure*, mmHg	106.2 ± 7.4	109.5 ± 7.5	0.19
Diastolic blood pressure*, mmHg	56.6 ± 3.7	59.2 ± 5.6	0.11
Central venous pressure*, mmHg	5.8 ± 2.5	4.8 ± 2.4	0.23
ECG V5 ST segment shift†, mV	0.1; 0.1–0.2	0.1; 0.1–0.2	0.69
Conversion, n	0 (0)	0 (0)	1.00
shunt introduction, n	0 (0)	17 (100)	

* Values are expressed as mean ± SD.

† Values are expressed as median; 95%LCL-95%UCL.

Values in parentheses are percentages.

TO = tournique occlusion; ILS = intraluminal-left anterior descending shunt; ECG = electrocardiogram; SD = standard deviation; LCL = lower confidence limit; UCL = upper confidence limit.

### cTnT analysis

**Figure 1:**
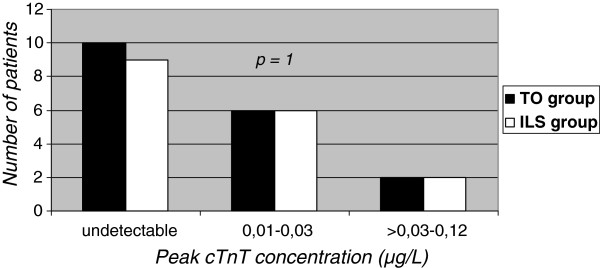
**Distribution of peak cardiac troponin T serum concentration postoperatively.** cTnT = cardiac troponin T; TO = tournique occlusion; ILS = intraluminal-left anterior descending shunt.

All patients included in the analysis had values of preoperative cTnT serum concentrations below LLD (<0.01 μg/L). Postoperatively, six patients from TO group (33.3%) and six patients from group ILS group (35.3%) were above the 99th-percentile (0.01 μg/L). Two patients from each group (TO group 11.1% and ILS group 11.8%) had peak values above the 10-% CV cutoff (0.03 μg/L) but did not exceed its quintiple diagnostic for coronary artery bypass grafting (CABG)-related MI. These four patients were indicative of peri-procedural myocardial necrosis. Distribution of the patients with cTnT values above the 99th-percentile and above the 10-% CV cutoff were similar, the groups did not differ (*p* = 1) from each other (Figure 1).

### Perioperative ECG V5 ST segment shift and cTnT

There were only two patients with perioperative ECG V5 ST segment shift above 1 mm in TO group (ST segment elevations 1.3 mV and 1.4 mV, anastomotis time 13 min. and 7 min.) with postoperative cTnT below LLD (<0.01 μg/L). Perioperative ECG ST-segment alterations of more than 1 mm was observed in only one patient from ILS group (ST segment elevation of 1.1 mV). There was prolonged shunt introduction (at about 3 min.) in this patient because of unfavourable anatomy of the LAD. Although the ST-segment elevation gradually vanished after shunt introduction, this patient had highest postoperative peak cTnT among all patients (0.12 μg/L) without further clinical consequences. Reversible perioperative ECG ST segment shifts were similar (*p* = 0.69) in both groups (Table 2).

### Post-operative results

Early mortality was zero. There were no twelve lead ECGs changes after surgery in our patients. There were no procedure-related myocardial infarctions and no neurologic, renal, pulmonary, wound or other serious complications observed. Rethoracotomy for bleeding was required in one patient from TO group. Discrete signs of low cardiac output (LCO) with catecholamines dependency of > 24 hours (< 0.05 μg/kg/min) occured in two patients from ILS group and in 4 patients from TO group (*p* = 0.66). Five of these patients had postoperative cTnT level below 10-% CV cutoff, one patient from TO group had peak cTnT level 0.078 μg/L. Additional postoperative echocardiography did not reveal new patological findings in these LCO patients. Post-operative data are summarized in Table 3.

**Table 3:** Postoperative complications

**Postoperative complications**	**TO group, n = 18**	**ILS group, n = 17**	***p*-value**
Blood losses†, ml	325; 300–450	350; 300–450	0.91
Rethoracotomy for bleeding, n	1 (5.6)	0 (0)	1.00
New atrial fibrillation, n	4 (22.2)	4 (23.5)	1.00
Low cardiac output			
IABP necessary, n	0 (0)	0 (0)	1.00
Prolonged catecholamines > 24 hours, n	4 (22.2)	2 (11.8)	0.66
Pleural effusion, n	1 (5.6)	0 (0)	1.00

† Values are expressed as median; 95%LCL-95%UCL.

Values in parentheses are percentages.

TO = tournique occlusion; ILS = intraluminal-left anterior descending shunt; IABP = intraaortic balloon pump; LCL = lower confidence limit; UCL = upper confidence limit.

## Discussion

Troponin remains the biomarker of choice for detection of cardiac injury. Troponin assays are more sensitive and more specific than CK-MB assays [16]. For all intents and purposes, cTnI and cTnT provide comparable information, except in patients with renal failure. Monitoring cardiac biomarkers are prerequisites to improve strategies for myocardial protection and surgical approaches [17].

Gürbüz at al. demonstrated in a small patient cohort that patients with isolated LAD lesions undergoing off-pump coronary artery bypass grafting (OPCAB) with intraluminal shunt revealed statistically less troponin I (cTnI) compared to no-shunt patients (*p* = 0.003). The observed discrete cTnT leaks in both groups may originate from “cytosolic pool“(unbound troponin) or from contractile apparatus in myofibrils (structurally bound troponin) [18]. The authors recommend the consequent use of an intraluminal shunt in OPCAB [7].

This was confirmed by studying OPCAB patients with multivessel disease (MVD) and moderate left ventricular dysfunction [19].

To our knowledge there are no published results in the literature regarding any OPCAB or MIDCAB studies comparing intraluminal shunting and tournique occlusion by means of cTnT during isolated revascularisation of the LAD. Accordingly any comparison of our results with above mentioned studies remains difficult. In contrast to the currently used cTnT assay, the approximately 10–20 cTnI immunoassays that have been developed use different antibodies directed against different epitopes and different calibrators and control materials [20,21]. However, contrary of the Gürbüz study, we could not confirm protective effect of the intraluminal shunting on myocardial damage in our MIDCAB patients. Gumm et al. examined the effect of risk area size on collateral resistance and ischemic region perfusion during LAD occlusion at different sites. They concluded, that small risk areas have significantly lower collateral resistance and receive more collateral flow per mass of tissue compared to large risk areas [22]. A potential explanation to the differing results of Gürbüz compared to our study might be the fact that the anastomotic site of the LAD in MIDCAB is placed more peripherally than in OPCAB.

At this time the discussion whether intraluminal-LAD shunting can provide a minimal blood flow for adequate myocardial protection, especially shunts of lower diameter remains controversal [23].

There are several concerns regarding intraluminal shunts versus tournique occlusion technique in off-pump revascularization. Zimarino et al. used intraoperative transoesophageal echocardiography (TEE) to assess the effects of prolonged LAD (TIMI flow 0–3) occlusion on myocardial dysfunction during MIDCAB, and evaluated the impact of myocardial ischemia on long-term outcome [5]. If the duration of ischemia of the LAD territory is limited to 30 minutes no apparent persistent wall motion abnormalities were observed. He concluded, that MVD, but not perioperative ischemia or stunning, predicts long-term event-free survival. Menon et al. tested the effectiveness of temporary intraluminal shunting during MIDCAB by using Swan-Ganz catheter and TEE. They concluded that shunts prevent systolic dysfunction and suggests an improvement of early graft patency and low reintervention rate within the first 6 postoperative months [3]. Based on intra- and postoperative angiography together with intraoperative TEE, Bergsland et al. stated that intracoronary shunts prevent ischemia (*p* = 0.004) during grafting of the LAD in OPCAB and provides satisfactory immediate- and short-term graft patency [4]. Nearly 30% of all patients developed de-novo LAD lesions (mostly proximal) near the anastomosis suggesting, that tournique occlusion even temporarily to permit shunt insertion, is traumatic to the coronary artery. The study should encourage more OPCAB surgeons to shunt routinely and avoid any proximal and distal tourniques.

Wippermann et al. have shown in an animal study that intraluminal shunts cause only moderate trauma to the vessel and may therefore be superior regarding acute and chronic intimal integrity in contrast to tournique occlusion [11,12]. In contrast, some investigators revealed that intracoronary shunts might create a greater degree of endothelial dysfunction compared to extravascular devices such as bulldog clamps or double-looped Gore-Tex suture snaring. They recommended to avoid intraluminal shunts [13-15,24]. Both external occlusion devices and intraluminal shunts may lead to target coronary artery occlusions, septal myocardial infarction, and distal embolization with atheromatous debris into the coronary circulation in some cases [25,26].

However, occlusion time of more than 30 minutes is known to potentially induce wall motion abnormalities and arrhythmias [6]. Accordingly in cases of repeated or difficult anastomosis an intracoronary shunt might be helpful and recommended.

### Limitations of the study

The inability to reliably quantify cTn in very low concentrations complicates a thorough data analysis. The inadequacy of the current commercially available cTnT assay (fourth generation) to distinguish potentially discrete differences in myocardial damage between techniques in MIDCAB is attributed both to the LLD being higher than reference values and to assay imprecision (i.e., CV) being >10% at the 99th-percentile value of the reference population. The application of recently developed highly sensitive cTn immunoassays may help to resolve this problem [20]. In general a study population with a larger size are required to obtain more accurate results.

## Conclusion

Safety of tournique occlusion or intraluminal shunting technique remains still controversial. Since 1997 we have used proximal and distal snares during MIDCAB at our department. After clinical introduction of shunts we have changed our strategy and used shunts routinely. As they can be occasionally difficult to insert or might displace during completion of the anastomosis we critically discussed tournique of the native vessel as an alternative to ensure good visualization of the anastomotic site. The results of our study confirmed this hypothesis by revealing no protective effect of intraluminal shunting on myocardial damage compared to tournique occlusion. Accordingly we changed our strategy again keeping it up the surgeon's discretion which method to prefer to achieve a bloodless field in grafting of the non-occluded LAD in MIDCAB.

## Abbreviations

MIDCAB: Minimally invasive direct coronary artery bypass grafting; LAD: Left anterior descending; TO: Tournique occlusion; cT: Cardiac troponin; CABG: Coronary artery bypass grafting; ILS: Intraluminal-LAD shunt; LV EF: Left ventricular ejection fraction; MI: Myocardial infarction; PCI: Percutaneous coronary intervention; LITA: Left internal thoracic artery; ECG: Electrocardiogram; ICU: Intensive care unit; LLD: Lower limit of detection; CV: Coeficient of variation; SD: Standard deviation; LCL: Lower confidence limit; UCL: Upper confidence limit; LCO: Low cardiac output; CK-MB: Creatine kinase MB; OPCAB: Off-pump coronary artery bypass grafting; MVD: Multivessel disease; TEE: Transoesophageal echocardiography.

## Competing interests

The authors declare that they have no competing interests.

## Authors’contributions

ZS and JH have made substantial contributions to conception, design and accquisition of data. JV has been involved in drafting the manuscript. RP has made substantial contributions to interpretation of data. EC has made analysis of data. UAS has given final approval of the version to be published. All authors have read the final manuscript.

## Authors’ information

ZS is cardiac surgeon and PhD student. A substantial part of research for his PhD thesis was on using shunt or occlusion in MIDCAB. JH, head of Dept.of Cardiac Surgery, is an experienced cardiothoracic surgeon.

## References

[B1] Lichtenberg A Klima U Paeschke H Pichlmaier M Ringes-Lichtenberg S Walles T Goerler H Haverich A Impact of multivessel coronary artery disease on outcome after isolated minimally invasive bypass grafting of the left anterior descending artery Ann Thorac Surg 2004 78 487 491 10.1016/j.athoracsur.2003.11.044 15276503

[B2] Jaffery Z Kowalski M Weaver WD Khanal S A meta-analysis of randomized control trials comparing minimally invasive direct coronary bypass grafting versus percutaneous coronary intervention for stenosis of the proximal left anterior descending artery Eur J Cardiothorac Surg 2007 31 691 697 10.1016/j.ejcts.2007.01.018 17300948

[B3] Menon AK Albes JM Oberhoff M Karsch KR Ziemer G Occlusion versus shunting during MIDCAB: effects on left ventricular function and quality of anastomosis Ann Thorac Surg 2002 73 1418 1423 10.1016/S0003-4975(02)03472-0 12022526

[B4] Bergsland J Lingaas PS Skulstad H Hol PK Halvorsen PS Andersen R Småstuen M Lundblad R Svennevig J Andersen K Fosse E Intracoronary shunt prevents ischemia in off-pump coronary artery bypass surgery Ann Thorac Surg 2009 87 1 54 60 10.1016/j.athoracsur.2008.08.032 19101268

[B5] Zimarino M Gallina S Di Fulvio M Di Mauro M Di Giammarco G De Caterina R Calafiore AM Intraoperative ischemia and long-term events after minimally invasive coronary surgery Ann Thorac Surg 2004 78 1 135 141 10.1016/j.athoracsur.2003.12.030 15223418

[B6] Dapunt OE Raji MR Jeschkeit S Dhein S Kuhn-Régnier F Südkamp M Fischer JH Mehlhorn U Intracoronary shunt insertion prevents myocardial stunning in a juvenile porcine MIDCAB model absent of coronary artery disease Eur J Cardiothorac Surg 1999 15 2 173 178 10.1016/S1010-7940(98)00290-5 10219550

[B7] Gürbüz A Emrecan B Yilik L Ozsöyler I Kestelli M Ozbek C Karahan N Intracoronary shunt reduces postoperative troponin leaks: a prospective randomized study Eur J Cardiothorac Surg 2006 29 2 186 189 10.1016/j.ejcts.2005.11.014 16376092

[B8] Thygesen K Alpert JS White HD Joint ESC/ACCF/AHA/WHF Task Force for the Redefinition of Myocardial Infarction: universal definition of myocardial infarction Circulation 2007 116 2634 2653 10.1161/CIRCULATIONAHA.107.187397 17951284

[B9] Lucchetti V Capasso F Caputo M Grimaldi G Capece M Brando G Caprio S Angelini GD Intracoronary shunt prevents left ventricular function impairment during beating heart coronary revascularization Eur J Cardiothorac Surg 1999 15 255 259 10.1016/S1010-7940(99)00005-6 10333019

[B10] Caputo M Chamberlain MH Ozalp F Underwood MJ Ciulli F Angelini GD Off-pump coronary operations can be safely taught to cardiothoracic trainees Ann Thorac Surg 2001 71 4 1215 1219 10.1016/S0003-4975(00)02686-2 11308162

[B11] Wippermann J Albes JM Brandes H Kosmehl H Bruhin R Wahlers T Acute effects of tourniquet occlusion and intraluminal shunts in beating heart surgery Eur J Cardiothorac Surg 2003 24 757 761 10.1016/S1010-7940(03)00520-7 14583309

[B12] Wippermann J Albes JM Bruhin R Hartrumpf M Vollandt R Kosmehl H Wahlers T Chronic ultrastructural effects of temporary intraluminal shunts in a porcine off-pump model Ann Thorac Surg 2004 78 2 543 548 10.1016/j.athoracsur.2004.02.099 15276516

[B13] Perrault LP Menasché P Bidouard JP Jacquemin C Villeneuve N Vilaine JP Vanhoutte PM Snaring of the target vessel in less invasive bypass operations does not cause endothelial disfunction Ann Thorac Surg 1997 63 751 755 10.1016/S0003-4975(96)01118-6 9066396

[B14] Hangler H Mueller L Ruttmann E Antretter H Pfaller K Shunt or snare: coronary endothelial damage due to hemostatic devices for beating heart coronary surgery Ann Thorac Surg 2008 86 6 1873 1877 10.1016/j.athoracsur.2008.06.047 19022000

[B15] Vural AH Yalcinkaya S Türk T Yümün G Gül N Yalcinkaya U Kaya M Ozyazicioğlu A Intracoronary shunt versus bulldog clamp in off-pump bypass surgery. Endothelial trauma: shunt versus clamp J Surg Res 2008 150 2 261 265 10.1016/j.jss.2007.12.774 18262555

[B16] Klein G Kampmann M Baum H Rauscher T Vukovic T Hallermayer K Rehner H Müller-Bardorff M Katus HA Clinical performance of the new cardiac markers troponin T and CK-MB on the Elecsys 2010. A multicentre evaluation Wien Klin Wochenschr 1998 110 Suppl 3 40 51 9677671

[B17] Babuin L Jaffe AS Troponin T The biomarker of choise for the detection of cardiac injury CMAJ 2005 173 10 1191 1202 10.1503/cmaj/051291 16275971 PMC1277047

[B18] Bleier J Vorderwinkler KP Falkensammer J Mair P Dapunt O Puschendorf B Mair J Different intracellular compartmentations of cardiac troponins and myosin heavy chains: a causal connection to their different early release after myocardial damage Clin Chem 1998 44 9 1912 1918 9732976

[B19] Emmiler M Kocogullari CU Ela Y Cekirdekci A Influence of intracoronary shunt on myocardial damage: a prospective randomized study Eur J Cardiothorac Surg 2008 34 5 1000 1004 10.1016/j.ejcts.2008.08.002 18783960

[B20] Mingels A Jacobs L Michielsen E Swaanenburg J Wodzig W van Dieijen-Visser M Reference population and marathon runner sera assessed by highly sensitive cardiac troponin T and commercial cardiac troponin T and I assays Clin Chem 2009 55 1 101 108 18988757 10.1373/clinchem.2008.106427

[B21] Apple FS Jesse RL Newby LK Wu AH Christenson RH Cannon CP Francis G Morrow DA Ravkilde J Storrow AB Tang W Jaffe AS Mair J Ordonez-Llanos J Pagani F Panteghini M Tate J IFCC Comittee on Standardization of Markers of Cardiac Damage National Academy of Clinical Biochemistry. National Academy of Clinical Biochemistry and IFCC Committee for Standardization of Markers of Cardiac Damage Laboratory Medicine Practice Guidelines: analytical issues for biochemical markers of acute coronary syndromes Clin Chem 2007 53 547 551 17384000 10.1373/clinchem.2006.084715

[B22] Gumm DC Cooper SM Thompson SB Marcus ML Harrison DG Influence of risk area size and location on native collateral resistance and ischemic zone perfusion Am J Physiol 1988 254 3 Pt 2 H473 H480 3348425 10.1152/ajpheart.1988.254.3.H473

[B23] Grünenfelder J Comber M Lachat M Leskosek B Turina M Zünd G Validation of Intracoronary Shunt Flow Measurements for Off-Pump Coronary Artery Bypass Operations Heart Surg Forum 2004 7 1 26 30 14980845

[B24] Chavanon O Perrault LP Menasche P Carrier M Vanhoutte PM Endothelial effects of hemostatic devices for continuous cardioplegia or minimally invasive operations Ann Thorac Surg 1999 68 1118 1120 10.1016/S0003-4975(99)00884-X 10510032

[B25] Izzat MB Yim AP El-Zufari MH Snaring of a coronary artery causing distal atheroma embolization Ann Thorac Surg 1998 66 1806 1808 10.1016/S0003-4975(98)00929-1 9875799

[B26] Izutani H Gill IS Acute graft failure caused by an intracoronary shunt in minimally invasive direct coronary artery bypass grafting J Thorac Cardiovasc Surg 2003 125 3 723 724 10.1067/mtc.2003.142 12658217

